# Solute carrier family 12 member 8 (SLC12A8) is a potential biomarker and related to tumor immune cell infiltration in bladder cancer

**DOI:** 10.1080/21655979.2021.1962485

**Published:** 2021-08-09

**Authors:** Qian Zhang, Yunen Liu, Peng Chen, Xiuyun Shi, Ying Liu, Lin Shi, Peifang Cong, Shun Mao, Cangci Tong, Cheng Du, Mingxiao Hou

**Affiliations:** a College of Medicine and Biological Information Engineering, Northeastern University, Shenyang, Liaoning, P.R. China; b Emergency Medicine Department of General Hospital of Northern Theater Command, Laboratory of Rescue Center of Severe Wound and Trauma PLA, Shenyang, Liaoning, P.R. China; c Department of Oncology, General Hospital of Northern Theater Command, Shenyang, Liaoning, P.R. China; d Department of Urology, General Hospital of Northern Theater Command, Shenyang, Liaoning, P.R. China

**Keywords:** SLC12A8, bladder cancer, tcga, cibersort, timer2.0, immune infiltration, immune checkpoint inhibitor

## Abstract

The solute carrier family has been reported to play critical roles in the progression of several cancers; however, the relationship between solute carrier family 12 member 8 (SLC12A8) and bladder cancer (BC) has not been clearly confirmed. This study explores the prognostic value of SLC12A8 for BC and its correlation with immune cell infiltration. We found that the expression of SLC12A8 mRNA was significantly overexpressed in BC tissues compared with noncancerous tissues in multiple public databases, and the result was validated using real-time PCR and immunohistochemistry (IHC). The Kaplan-Meier method and Cox proportional hazards models were used to evaluate the prognostic value of SLC12A8 for BC. The high expression of SLC12A8 led to a shorter overall survival time and was an unfavorable prognostic biomarker for BC. The mechanisms of SLC12A8 promoting tumorigenesis were investigated by Gene Set Enrichment Analysis (GSEA). Moreover, the correlations of SLC12A8 expression with the tumor-infiltrating immune cells (TICs) in BC were explored using TIMER 2.0 and CIBERSORT. SLC12A8 was associated with CD4+ T cells, dendritic cells, neutrophils, and macrophages infiltration. The expression of SLC12A8 was positively correlated with crucial immune checkpoint molecules. In conclusion, SLC12A8 might be an unfavorable prognostic biomarker in BC related to tumor immune cell infiltration.

## Introduction

The incidence of BC ranks 10th, and mortality ranks 13th in cancers globally; among men, BC ranks 7th in incidence and 9th in mortality [1–3]. Neoadjuvant or adjuvant chemotherapy improves survival and reduces the recurrence rate of muscle-invasive BC after radical cystectomy [4–6]. Nevertheless, 5-year survival from metastatic BC is only 5% [7]. Compared with traditional chemotherapy, immunotherapy has good efficacy in advanced or metastatic urothelial carcinoma [8]. Several immune checkpoint inhibitors (ICIs) have been approved as second-line treatments for patients that have progressed during or after previous platinum-based chemotherapy. Atezolizumab and pembrolizumab also received approval as first-line treatments for patients ineligible to receive cisplatin [9]. However, at present, immunotherapy still presents problems such as low response rates and high prices. Only a few patients can benefit from ICIs treatment. By contrast, many patients have an unsatisfactory response to ICIs, which may lead to economic waste. For these reasons, there is an urgent need to identify new predictors in cancer patients and select potential beneficiaries of immunotherapy to achieve precision therapy [10]. The outcomes and anti-tumor responses of immunotherapy depend on T cell infiltration [11]. In addition, TICs play a crucial role in tumor progression. Therefore, we investigated the abundance of immune cells in BC samples and correlated with the expression of SLC12A8.

SLC12A8 belongs to the solute carrier (SLC) transporters family. The most significant transporters in the body have essential roles in regulating the transport of substances inside and outside cells [12–16]. A recent study demonstrated that SLC12A8 is a nicotinamide mononucleotide transporter [17]. Although an association between the SLC family and the progression of urinary tract tumors has been mentioned, there is a lack of sufficient evidence to establish a correlation between SLC12A8 and BC prognosis [18,19].

This study aimed to explored the prognostic value of SLC12A8 in BC using the public database TCGA and GES13507. To further confirm the clinical diagnostic value of SLC12A8, qPCR and immunohistochemistry (IHC) methods were performed in the collected clinical tissue samples. The possible molecular mechanisms and signal pathways by which SLC12A8 participates in BC were analyzed. Finally, Timer2.0 and CIBERSORT were used to evaluate the effect of SLC12A8 on immune cell infiltration and its relationship with immune checkpoint protein expression in BC.

## Materials & methods

### Databases

TCGA-BLCA includes gene profile data of 414 BC samples and 19 noncancerous samples. Oncomine and GES13507 were used to verify the differential expression of SLC12A8 mRNA in BC tissues (n = 188) and normal tissues (n = 68) [20,21]. Clinical and pathological data of BC patients were obtained from a TCGA-BLCA cohort (n = 427) and the GSE13507 cohort (n = 165). Patients with incomplete clinical information were excluded.

### Evaluation of the prognostic value of SLC12A8 in BC

To further evaluate the prognostic value of SLC12A8 in BC, patients were divided into two groups according to the median expression of SLC12A8. The Kaplan-Meier method was used to explore 5-years overall survival or cancer-specific cancer specific survival time in TCGA and GSE13507. Univariate and multivariate Cox regressions were used to evaluate proportional hazards for overall survival [22]. The patients’ risk scores were further evaluated, and the risk receiver operating characteristic (ROC) curves were plotted.

### Gene Ontology (GO) and Kyoto Encyclopedia of Genes and Genomes (KEGG) pathway analysis

Genes that are co-expressed with SLC12A8 were obtained from the Multi Experiment Matrix (MEM) (https://biit.cs.ut.ee/mem/index.cgi), a gene expression query, and visualization tool [23]. To explore the possible mechanism of SLC12A8 involvement in BC, co-expressed genes were used to conduct GO and KEGG pathway analysis [24,25]. A protein-protein interaction (PPI) network analysis of SLC12A8 was conducted using STRING (https://string‐db.org/) [26].

### Gene Set Enrichment Analysis

GSEA v3.0 (http://www.broad.mit.edu/gsea/) was used to determine significant and concordant differences between a set of biological processes or signaling pathway genes from the Molecular Signatures Database (MsigDB) and SLC12A8 high expression groups based on TCGA with the cut‐off criteria of FDR < 0.25 and nominal p < 0.05 [27].

### Patients and characteristics

The study included 29 pairs of BC tissues and normal adjacent tissues from the General Hospital of Northern Theater Command between November 2019 and June 2020. Patient age ranged from 48 to 84 years, with a mean of 69.6 years, including 27 males and two females. All patients underwent radical cystectomy in the Department of Urology without chemotherapy. The tissues and paraffin sections used in this study were the remaining tissues and paraffin sections reserved in the pathology department. The General Hospital approved the study of Northern Theater Command (Shenyang, Liaoning, P.R. China), and all patients gave verbal consent. RB approval number: Y (2021) 039.

### Cell culture

One normal human urinary tract epithelial SV-HUC-1 and five urinary BC cell lines (T24, UMUC3,5637, J82, and EJ-1) were obtained from FuHeng Biology. T24, 5637, and EJ-1 cells were cultured in RPMI 1640 medium, UMUC3 was cultured in DMEM medium, J82 was cultured in MEM medium, and SV-HUC-1 was cultured in F-12 K medium. All media contained 10% fetal bovine serum. The cell culture conditions were 37 °C and 5% CO_2_.

### Real-Time PCR

Total mRNA of BC tissues or cell lines were extracted using TRIzol (Invitrogen, #119,706). Reverse transcription was performed using Fasting gDNA Dispelling RT SuperMix Kit (TIANGEN BIOTECH, #KR118). The reverse transcription conditions were as follows: 42 °C for 15 min, 95 °C for 3 min. qPCR was performed using the TL988 Real-Time PCR System (TIANLONG TECHNOLOGY). According to SYBR Green Kit (BIO-RAD, #1,725,121), the reaction conditions were 95 °C for 60 sec, 95 °C for 10 sec, and 63 °C for 30 sec by 40 cycles. The sequence of SLC12A8 primer pairs were 5′-AGAAAGCTCCCAGTTACGGC-3′(forward), and 5′-CTGGGCTGGCTACTCTCAAG-3′ (reverse) and GAPDH were 5′-AGTCCACTGGCGTCTTCAC-3′ (forward) and 5′-GAGGCATTGCTGATGATCTTGA-3′ (reverse). 2 ^ΔΔCT^ method was used to quantify relative SLC12A8 mRNA expression [28].

## SLC12A8 IHC

The protein expression levels of SLC12A8 in tissue samples were determined using IHC. Briefly, 4-μm paraffin tissue chips were baked at 65 °C for 1 hour before dewaxing. The staining procedure was operated according to the IHC kit protocol (Maxim Biotechnology) using the SLC12A8 antibody (Biorbyt, #orb317876,1:200 dilution). The sections were subjected to DAB color development, hematoxylin staining, dehydration, and sealing. The sections were taken photographs under a microscope with ×100 and ×200 magnification. IHC profiler, a plug-in of Image J, was used to analyze the immunohistochemical results. The percentage of positive cells was divided into five grades according to the following scoring rules: less than 10% = 0, 10%–25% = 1, 26–50% = 2, 51–75% = 3 score, and more than 75% = 4. The staining intensity was divided into four grades: negative = 0, weak staining = 1, moderate staining = 2, and strong staining = 3. The positive proportion score was multiplied by the staining intensity score; according to the corresponding expression classification, 0–3 points indicated low expression and 4–12 points indicated high expression [29].

### Evaluation of immune infiltration

The proportion of 22 immune cells in BC samples were calculated using CIBERSORT [30,31]. TIMER2.0 provides a more comprehensive assessment of immune infiltration levels using seven state-of-the-art algorithms for TCGA [32]. It provides correlations between gene expression and the level of 20 cell immune infiltrates in various cancer types. We summarized the correlation between SLC12A8 expression and immune cell infiltrates in BC using all algorithms including TIMER, CIBERSORT, CIBERSORT-ABS, EPIC, QUANTISEQ, XCELL, and MPC-COUNTER provided in TIMER2.0 and displayed them with a heatmap.

### Correlation with immune checkpoints

To evaluate the response and therapeutic outcomes of immune checkpoints through SLC12A8 gene expression, we further analyzed the correlation between SLC12A8 expression and important immune checkpoints gene expression according to TCGA-BLCA expression profile data [33]. TIMER2.0 provided Pearson correlation coefficients between genes.

### Statistical analysis

Statistical processing and analysis were performed using R (version 3.6.2) and SPSS Statistics 26.0 in this study. Independent T-tests or non-parametric tests were used to compare SLC12A8 mRNA expression between groups. The chi-squared test was to determine relevance between clinicopathologic characteristics and SLC12A8 expression levels [34]. The Kaplan–Meier method was used for survival analysis. A Cox analysis regression model was used to assess independent prognostic factors. ROC curves were used to estimate the diagnosis values.

### Results

The expression of the SLC12A8 gene was significantly increased in BC tissues identified by bioinformatics analysis and experiment determination. Overexpression of SLC12A8 was correlated with poor outcome as an unfavorable biomarker in BC. We also found that SLC12A8 was related to immune cell infiltration and positively correlated with crucial immune checkpoint molecules in BC. The pathway analysis indicated the mechanism of SLC12A8 involved in the tumorigenesis.

### Overexpression of SLC12A8 mRNA in BC

**Figure 1.**
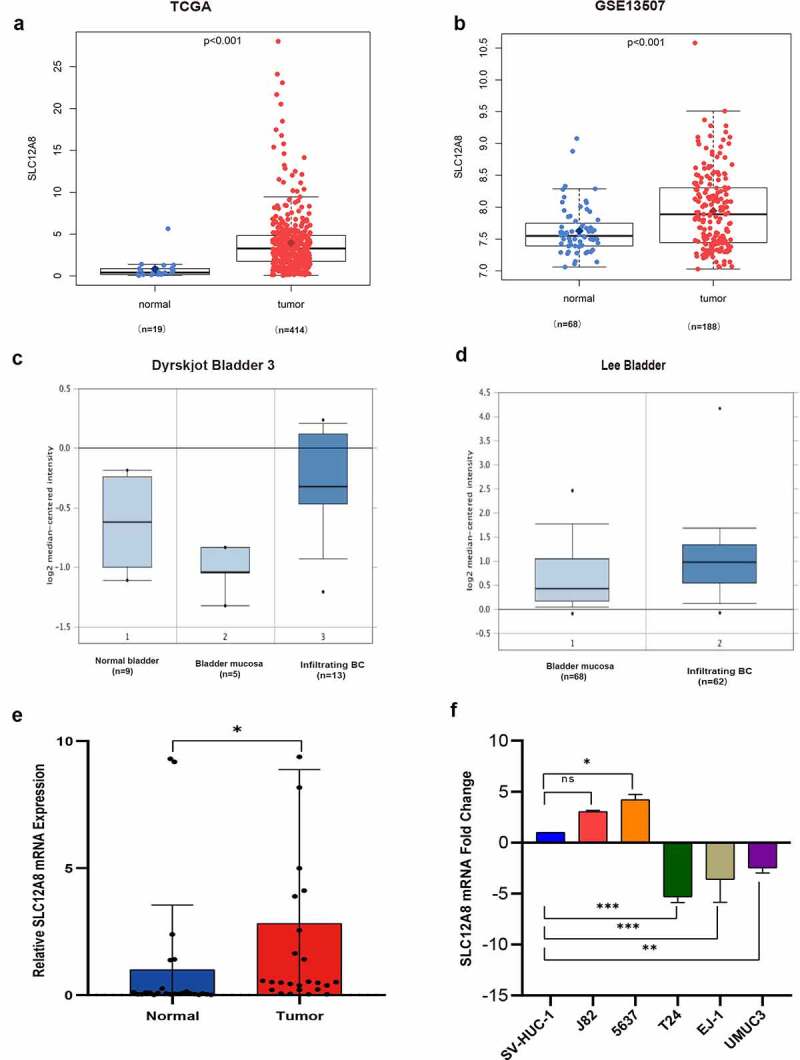
**Different expression levels of SLC12A8 mRNA between normal tissues and BC tissues**. (a) The expression of SLC12A8 mRNA in noncancerous tissues (n = 19) and BC tissues (n = 414) from TCGA. (b) The expression of SLC12A8 mRNA in normal tissues (n = 68) and BC tissues (n = 188) from GSE13,507. (c) The expression of SLC12A8 mRNA in normal bladder tissues (N = 9), bladder mucosa (N = 5) and infiltrating BC tissues (N = 13) from Dyrskjot Bladder 3 dataset in Oncomine. (d) The expression of SLC12A8 mRNA in normal bladder tissues (N = 48) and infiltrating BC tissues. *P < 0.05, **P < 0.01, *** P < 0.001

The expression levels of SLC12A8 mRNA in normal and BC tissues were evaluated using BC gene expression profiles from TCGA-BLCA. The expression of SLC12A8 mRNA was significantly greater in BC tissues than in normal tissues (P < 0.001 Figure 1a). A greater expression of SLC12A8 mRNA in BC tissues than normal tissues was validated using the GSE13507 dataset (Figure 1b). The Oncomine database indicated that SLC12A8 mRNA levels were elevated in infiltrating urothelial bladder tissues than those in normal tissues from two datasets (Figure 1c, 1d). qPCR analysis of 25 groups of clinical tissues confirmed that the expression levels of SLC12A8 in tumor tissues were significantly greater (Figure 1e). However, qPCR in the cell lines showed that, compared with normal bladder epithelial cells (SV-HUC-1), expression levels of SLC12A8 mRNA in J82 and 5637 were greater, T24, EJ-1, and UMUC3 were lower (figure 1f).

### Kaplan-Meier survival analysis of SLC12A8 in BC

**Figure 2.**
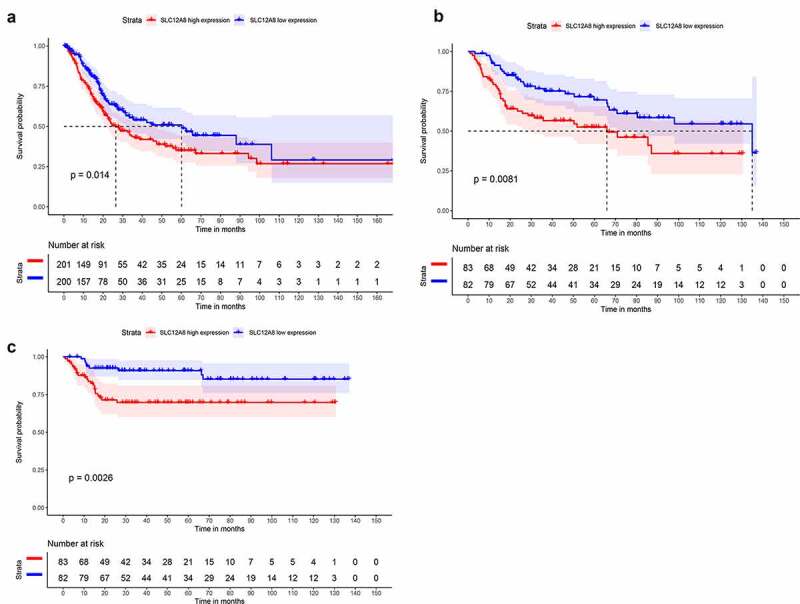
**Survival plots of SLC12A8 in TCGA cohort and in GSE13,507 cohort**. (a) The relationship between the expression of SLC12A8 mRNA and the overall survival of BC patients from TCGA cohort. (b) The relationship between the expression of SLC12A8 mRNA and the overall survival of BC patients from GSE13507 cohort. (c) The relationship between the expression of SLC12A8 mRNA and the cancer specific survival of BC patients from GSE13507 cohort

To evaluate the prognostic value of SLC12A8 in BC, the survival data of patients in TCGA and GSE13507 were analyzed. BC patients with high expression of SLC12A8 had remarkably shorter overall survival than those in the low expression group both in TCGA (P = 0.014, Figure 2a) and GES13507 cohort (P = 0.0081, Figure 2b). Consistently, SLC12A8 overexpression was considered a prognostic factor of cancer-specific survival in GSE13507. (P = 0.007, Figure 2c).

### Correlation between SLC12A8 mRNA expression and the clinical characteristics of BC patients

Clinical characteristics of 427 BC patients from TCGA-BLCA cohort and 165 patients from the GSE13507 cohort were associated with high and low SLC12A8 expression. SLC12A8 mRNA expression was significantly associated with age (p < 0.01), pathological stage (p < 0.001), histological grade (p < 0.001), N stage (p < 0.001) and M stage (p < 0.001) in TCGA-BLCA cohort. In the GSE13507 dataset, the expression of SLC12A8 was closely associated with age (p < 0.001), pathological grade (p < 0.001), T stage (p < 0.001), and N stage (p < 0.05) (Table 1).

**Table 1.** Association between clinical characteristics of BC patients and SLC12A8 expression

clinical characters	TCGA cohort			GSE13507 cohort		
	SLC12A8	SLC12A8	p	SLC12A8	SLC12A8	P
	Low expression	High expression		Low expression	High expression	
	(n = 213)	(n = 214)		(n = 82)	(n = 83)	
Age			0.006			0.026
<65	91(42.7%)	64(29.9%)		36(43.90%)	23 (27.71%)	
≥65	122(57.3)	150(70.1%)		46(56.10%)	60 (72.29%)	
Gender			0.534			0.442
MALE	158(74.2%)	153(71.5%)		69(84.15%)	66(79.52%)	
FEMALE	55(25.8%)	61(28.5%)		13(15.85%)	17(20.48%)	
Pathological stage			<0.001			
Stage I&II	92(43.2%)	44(20.56%)		NA	NA	
Stage III&IV	120(56.3%)	169(78.97%)		NA	NA	
No data	1(0.47%)	1(=0.47%)		NA	NA	
Histological grade			<0.001			<0.001
Low grade	21(9.86%)	0(0.00%)		69(84.15%)	36(43.37%)	
High grade	192(90.14%)	211(98.60%)		13(15.85%)	47(56.63%)	
No data	0(0.00%)	3(1.40%)				
T stage			0.967			<0.001
T_0_-T_1_	2(0.94%)	2(0.93%)				
T_2_-T_4_	191(89.67%)	199(93.00%)		19(23.17%)	41(49.40%)	
No data	20(9.39%)	13(6.07%)		63(76.83%)	42(50.60%)	
N stage			<0.001			0.008
N_0_	141(66.20%)	107(50.00%)		79(96.34%)	68(81.93%)	
N_1_-N_3_	53(24.88%)	84(39.25%)		3(3.66%)	13(15.66%)	
No data	19(8.92%)	23(10.75%)			2(2.41%)	
M stage			0.044			0.45
M_0_	153(71.83%)	53(24.77%)		80(97.56%)	78(93.98%)	
M_1_	4(1.88%)	6(2.80%)		2(2.44%)	5(6.02%)	
No data	56(26.29%)	155(72.43%)				

### Cox univariable and multivariable analysis of overall survival among BC patients

**Figure 3.**
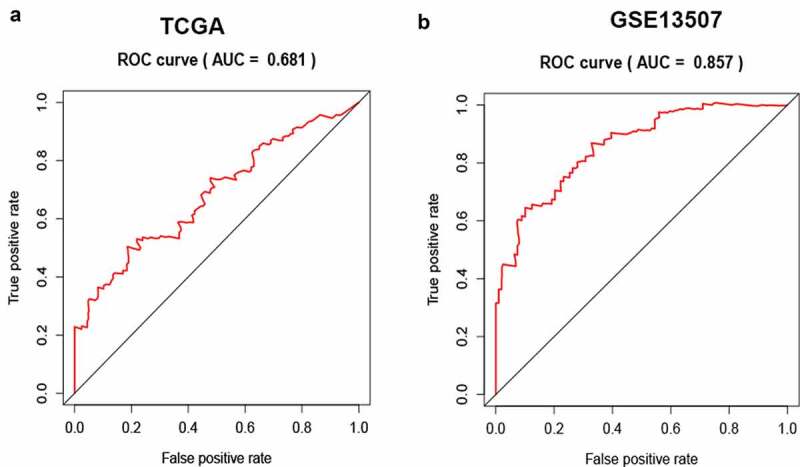
**Receiver operating characteristic (ROC) curve of SLC12A8 for predicting 5-years overall survival of BC patients**. Evaluation of the prognostic value of SLC12A8 using AUC in (a) TCGA cohort and in (b) GSE13507 cohort

In TCGA-BLCA cohort, Cox univariable analysis showed pathological stage (hazard ratio (HR) = 1.7618, p < 0.001), T stage (HR = 1.6423, p < 0.001), N stage (HR = 1.5524, p < 0.001), M stage (HR = 2,4975, p < 0.01) and SLC12A8 expression (HR = 1.5810, p < 0.001) influenced overall survival. Multivariable Cox analysis showed that only SLC12A8 expression (HR = 1.4185, p < 0.001) independently influenced overall survival among BC patients (Table 2). In the GSE13507 cohort based on univariate Cox analysis, SLC12A8 overexpression was predicted poor cancer-specific survival in BC patients (HR = 1.2338, p < 0.01); however, it was not an independent factor by multivariable Cox analysis (HR = 0.6537, p = 0.2715) (Table 3). To determine the diagnostic capacity of SLC12A8 expression as an unfavorable prognostic biomarker in BC, we calculated the area under the curve (AUC) values of 5-year survival risks using ROC curves. The AUCs were 0.681 and 0. 857 for the TCGA and GSE13507 cohorts, respectively (Figure 3a, 3b).

**Table 2.** Univariate and multivariate cox analysis of clinical characteristics for OS in TCGA cohort

Clinical Variables	TCGA cohort				TCGA cohort			
	Univariate analysis				Multivariate analysis			
	HR	HR.95L	HR.95H	P value	HR	HR.95L	HR.95H	P value
Gender	0.6457	0.3857	1.0808	0.096	0.6862	0.4013	1.1734	0.1689
Age	1.0245	0.999	1.0505	0.0596	1.0201	0.9936	1.0472	0.1379
Grade	3.7586	0.5167	27.3424	0.191	1.1678	0.1464	9.3138	0.8836
Stage	1.7618	1.2643	2.455	**0.0008**	1.0986	0.5417	2.2282	0.7944
M	2.4975	0.9971	6.2556	0.0507	1.3638	0.4632	4.0152	0.5733
N	1.5524	1.2057	1.9986	**0.0006**	1.2343	0.7487	2.0347	0.4091
T	1.6423	1.1447	2.3562	**0.0071**	1.3639	0.8317	2.2366	0.2189
SLC12A8	1.581	1.209	2.0675	**0.0008**	1.4185	1.0395	1.9355	**0.0275**

**Table 3.** Univariate and multivariate cox analysis of clinical characteristics for cancer specific survival in GSE13507 cohort

Clinical Variables	GSE 13507 cohort				GSE 13507 cohort			
	Univariate analysis				Multivariate analysis			
	HR	HR.95L	HR.95H	P value	HR	HR.95L	HR.95H	P value
Gender	0.4561	0.2097	0.9922	**0.0477**	0.6453	0.2686	1.5506	0.3275
Age	1.0526	1.0166	1.0899	**0.0039**	1.0924	1.0402	1.1473	**0.0004**
Grade	5.7791	2.6494	12.6058	**0.0000**	0.8197	0.3186	2.1091	0.6801
M	13.8632	5.552	34.616	**0.0000**	8.5178	2.32	31.2731	**0.0012**
N	10.328	4.9256	21.6557	**0.0000**	2.2339	0.8627	5.7845	0.0978
T	3.7044	2.6058	5.2663	**0.0000**	1.9891	1.1159	3.5454	**0.0197**
Invasiveness	24.1573	7.3165	79.7621	**0.0000**	4.9689	0.8543	28.901	0.0743
Progressive	6.1295	3.0161	12.4569	**0.0000**	6.416	2.4492	16.8071	**0.0002**
SLC12A8	2.0518	1.2338	3.412	**0.0056**	0.6537	0.3064	1.3946	0.2715

### IHC of SLC12A8 protein expression

**Figure 4.**
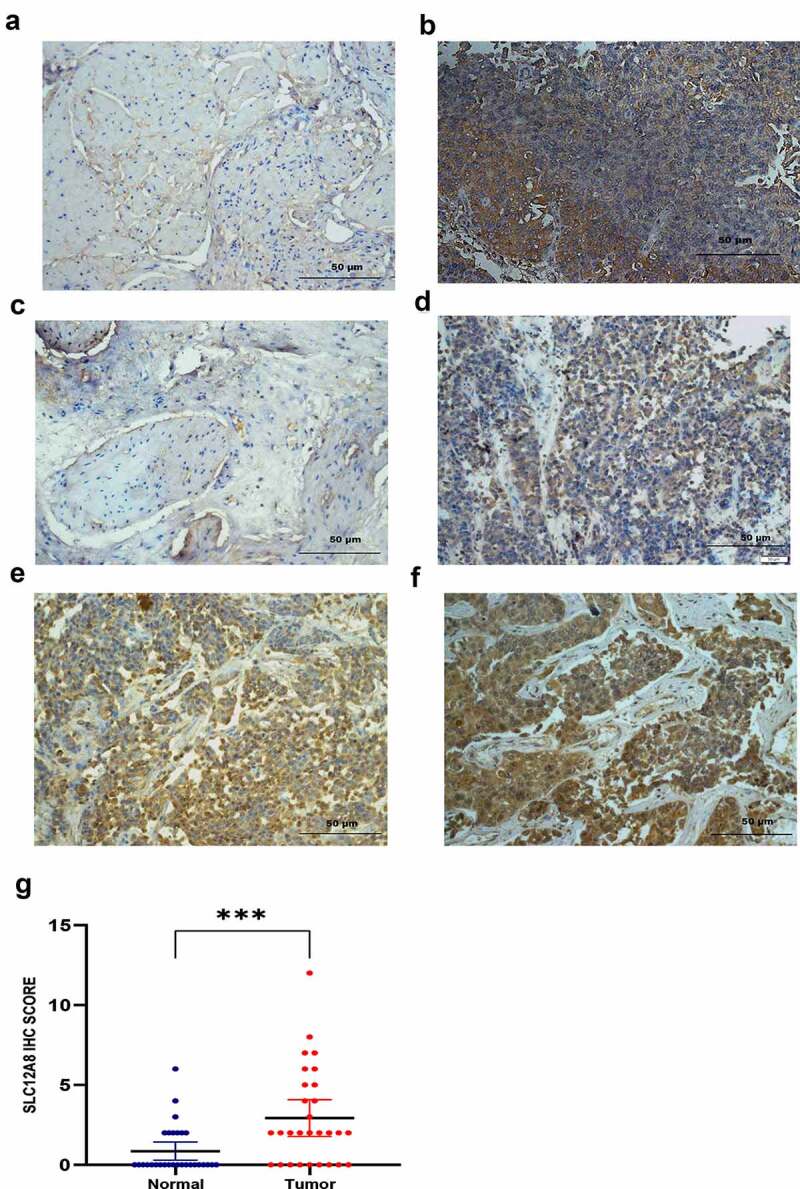
**SLC12A8 protein expression in tissues**. Representative images of SLC12A8 in adjacent tissues (a) and in bladder tumor tissues (b). Bladder tumor tissues were divided into four grades according to the staining intensity: negative (c), weak (d), moderate (e) and strong (f). Immunohistochemistry score of SLC12A8 in 29 pairs of bladder cancer and adjacent normal tissues (g). ***P < 0.001

IHC was performed on 29 paired BC tissues and adjacent normal tissues to examine SLC12A8 protein expression levels further. Representative IHC staining image indicated that SLC12A8 was located in the cytoplasmic and plasma membrane. IHC scores suggested that levels of protein expression of SLC12A8 in BC tissues were significantly higher than in adjacent tissues (Figure 4a, 4b). According to the pathological grade, the IHC staining of the low-grade group was negative (Figure 4c). In contrast, the staining of patients diagnosed as high-grade were weak (Figure 4d), moderate (Figure 4e), or strong (figure 4f). IHC staining scores of SLC12A8 in the BC group were significantly higher than in the normal group (P < 0.005) (Figure 4g).

### GO and KEGG pathway analysis of genes co-expressed with SLC12A8

**Figure 5.**
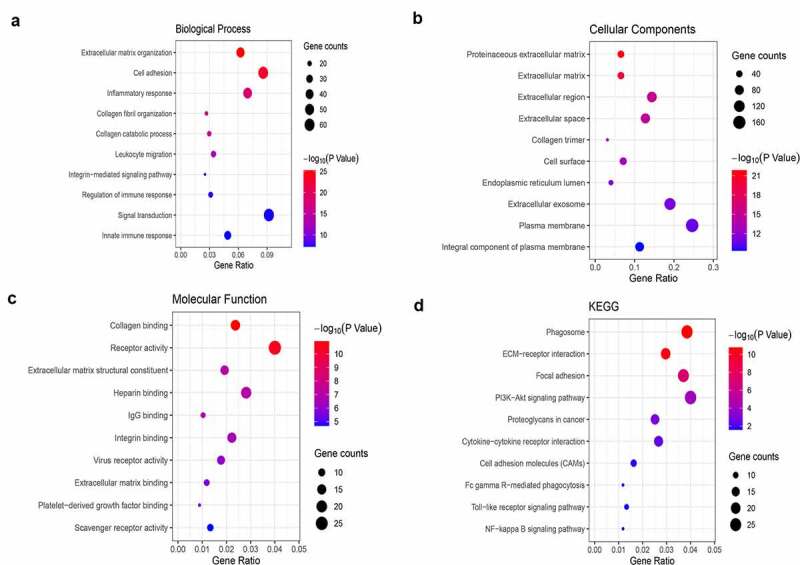
**GO and KEEG analysis of co-expression genes of SLC12A8**. (a) GO analysis for biological process. (b) GO analysis for cellular components. (c) GO analysis for molecular functions. (d) KEEG pathway analysis

The genes co-expressed with SLC12A8 were found in MEM and cBioPortal databases. There were 50 co-expressed genes of SLC12A8 in the MEM (P < 0.001) and cBioPortal databases. Spearman correlation coefficient > 0.4 was used as the criteria for inclusion (P < 0.001). The GO and KEGG analyses of 483 co-expressed genes after eliminating duplicates showed that they were enriched in extracellular matrix tissue, cell adhesion, inflammatory response, and signal transduction in terms of biological process (Figure 5a). The co-expressed genes were enriched in the collagen-containing extracellular matrix, endoplasmic reticulum cavity, focal adhesion, and cell-substrate adhesion (Figure 5b). The co-expressed genes were enriched in extracellular matrix structural constituents, glycosaminoglycan binding, and immunoglobulin binding (Figure 5c). The KEGG pathway indicated that co-expressed genes were enriched in the PI3K-Akt signaling pathway, extracellular matrix (ECM)-receptor interactions, proteoglycans in cancer, cytokine-cytokine receptor interactions, cell adhesion molecules, the toll-like receptor signaling pathway, the NF-kappa B signaling pathway, and focal adhesion (Figure 5d).

**Figure 6.**
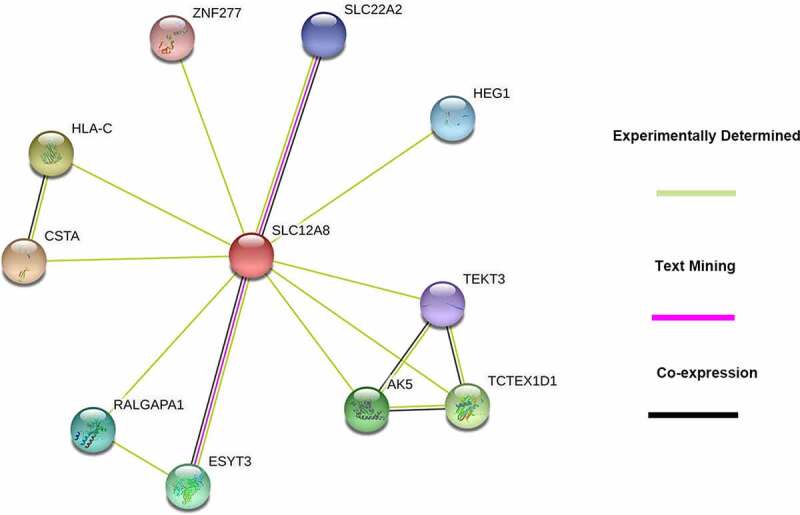
The PPI networks of SLC12A8 protein performed by STRING

The PPI network of SLC12A8 described by STRING to detect the interacting protein showed 11 nodes, 15 edges, and an average local clustering coefficient of 0.919 (Figure 6). Cystatin A, the maximum-score protein, mediates cell-cell adhesion in the lower levels of the epidermis. SLC22A2-mediated tubular uptake of organic compounds from circulation and ESYT3-associated lipid transport in cellular were proteins co-expressed with SLC12A8.

### SLC12A8 associated gene set enrichment in cancer

**Figure 7.**
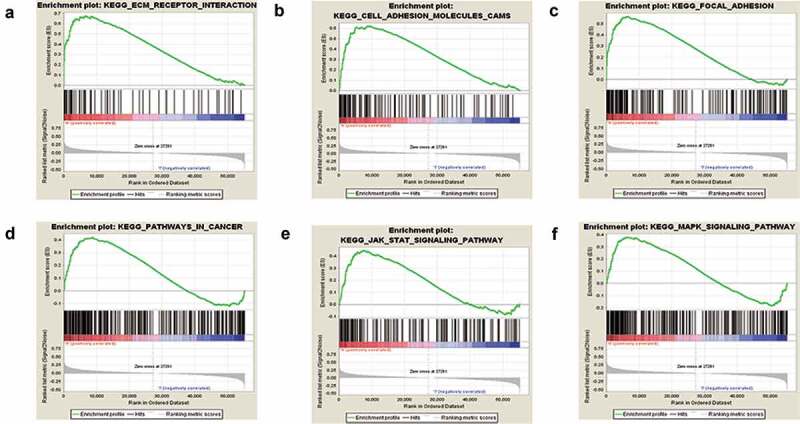
**Gene set enrichment analysis of associated genes with SLC12A8**.The results of GSEA analysis showed high expression of SLC12A8 were related the pathway of (a) ECM receptor interaction (b) Cell adhesion molecules cams (c) Focal adhesion. (d) Pathway in cancer (e) JAK-STAT signaling pathway and (f) MAPK signaling pathway

We used GSEA to identify associated genes enriched in response to SLC12A8 high expression based on TCGA-BLCA. The chemokine signaling pathway, cell adhesion molecules, ECM receptors, and the cancer pathway related to tumorigenesis, invasion, and metastasis were significantly enriched in the group of high SLC12A8 expression. GSEA analysis indicated that SLC12A8 might play an essential role in BC development (Figure 7).

### Correlation between SLC12A8 and TICs

**Figure 8.**
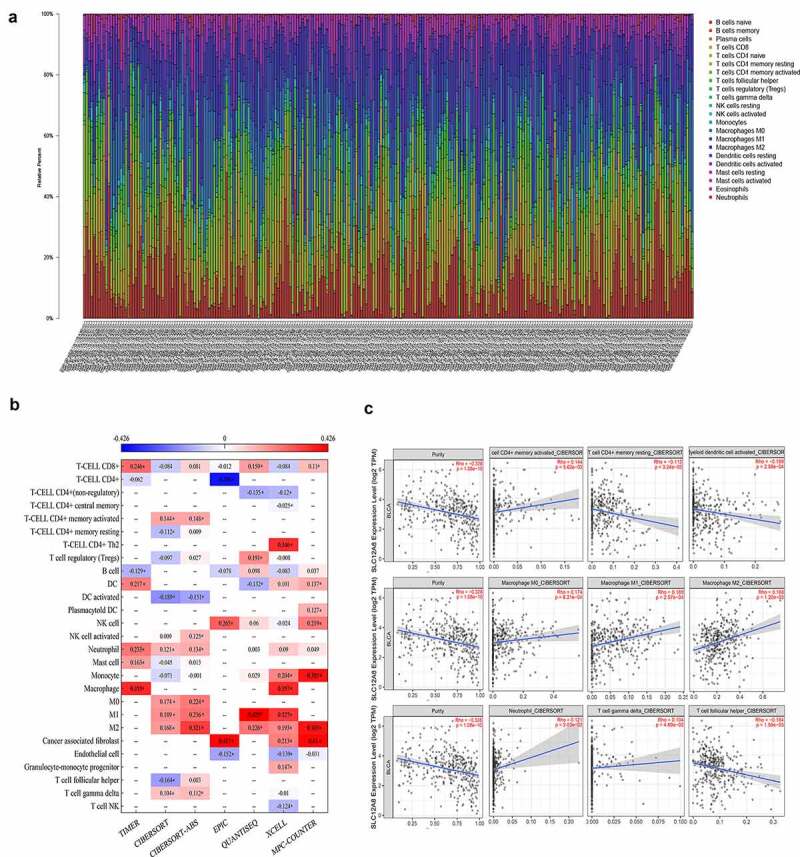
**The abundance of immune cells in BC samples and TICs correlation with SLC12A8 expression**. (a) Barplot showing the proportion of 22 immune cells in BC samples. (b) Heatmap showing the Spearman’s coefficient of TICs and SLC12A8 expression through seven different algorithms. (* p < 0.05) (c) Scatter plot showing the correlation of nine TICs with SLC12A8 expression in CIBERSORT (p < 0.05)

The compositions of 22 immune cells in BC samples calculated by CIBERSORT were filtered using the criterion P < 0.05, and the filtered results of 11 normal samples and 238 tumor samples were displayed using a barplot (Figure 8a). The seven algorithms possess varying accuracies for estimating different cell types; Timer 2.0 summarizes the results of these important algorithms. This study extracted the correlation between the SLC12A8 gene and TICs in patients with BC and drew the heat map (Figure 8b). According to the results of the CIBERSORT method, SLC12A8 expression was positively correlated with CD4+ memory activated T cells, neutrophil, M0, M1, M2 macrophage, and gamma delta T cells, and negatively correlated with resting CD4+ memory T cells, activated dendritic cells, and follicular helper T cells (Figure 8c).

### SLC12A8 was positively correlated with common immune checkpoints

**Figure 9.**
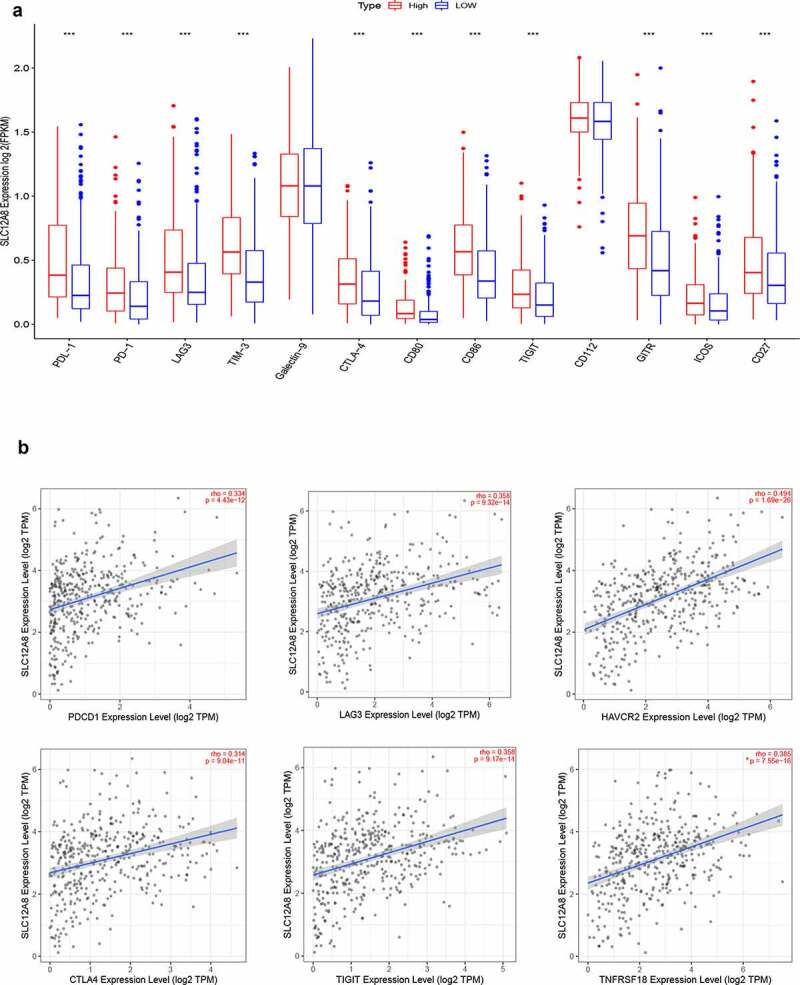
**The correlation of SLC12A8 expression with immune checkpoints**. (a) The expression of immunotherapy-related markers was evaluated in SLC12A8 high expression groups. (b) Spearman’s coefficient of immune checkpoints and SLC12A8 expression analyzed using TIMER2.0

The effect of gene expression level on the potential efficacy of immunotherapy was further investigated. We found that the expression of SLC12A8 was positively correlated with the expression of immunotherapy-related markers, PDL-1 (p < 0.001), CTLA-4 (p < 0.001), LAG-3 (p < 0.001), TIM-3 (p < 0.001) and TIGIT (p < 0.001), stimulatory checkpoint molecules, GITR (p < 0.001), ICOS (p < 0.001) and CD27 (p < 0.001) (Figure 9a). Spearman’s correlation analysis of SLC12A8 gene expression and major immune checkpoints were conducted using TIMER 2.0. SLC12A8 expression showed a positive correlation with major immune checkpoint molecules PD-L1 (PDCD1), LAG3, TIM-3 (Havcr2), CTLA-4, and TIGIT (Figure 9b).

### Discussion

Due to the lack of confirmed specific molecules, BC diagnosis and targeted therapy are greatly restricted [35]. Therefore, new therapeutic strategies and biomarkers are needed urgently. The SLC family is relevant to the genesis and progression of various cancers [36–38]. SLC transporters for essential nutrients may promote tumor occurrence; however, some subtypes inhibit tumors by increasing the accumulation of anti-tumor drugs in cells [39]. Also, SLC transporters can mediate immune cell homeostasis through transporters of immune cells [40,41].

Furthermore, SLC transporters regulate energy metabolism by glucose uptake mediation and then affect cell proliferation and tumor microenvironment [42,43]. SLC12A8 is a member of the SLC family that participates in various biological processes, including ion and nutrient delivery, cell energy metabolism and cell volume regulation, and drug delivery [44]. Whether the relationship between SLC12A8 and tumor is related to the mechanism of immunity, signal transduction, and tumor microenvironment has not been clarified.

In the present research, we used public cancer databases for data mining and found SLC12A8 mRNA overexpression in BC associated with poor prognosis. Real-time PCR further validated the finding in BC tissues and urinary tract cell lines. SLC12A8 mRNA expression was significantly elevated in BC tissues. However, the fold change of SLC12A8 mRNA in T24, UMUC3, and EJ-1 was negative, consistent with published data in Broad Institute Cancer Cell Line Encyclopedia (https://portals.broadinstitute.org/ccle). The expression of SLC12A8 protein was localized to the cell membrane. The staining of normal and low-grade tissues was almost negative, and the high-grade presented weak, moderate, or intense staining.

To further explore the possible mechanism of SLC12A8 in the progression of BC, we performed GO, KEGG, and PPI analyses. GO & KEGG pathway analysis and GSEA results revealed that SLC12A8 mainly affects tumor progression by acting on tumor-related pathways, including NF-kb, PI3K-Akt, and Toll-like receptors. Meanwhile, SLC12A8 may regulate ECM-receptor interaction, extracellular matrix organization, cell adhesion, and immunoglobulin binding to impact tumor microenvironment and immunity. Cystatin A, a protein that interacts with SLC12A8, is a putative tumor suppressor that modulates extracellular matrix remodeling, cell adhesion, tumor invasion, and metastasis [45,46]. By contrast, in some studies, cystatin A was considered a poor prognostic biomarker in pancreatic cancer, nasopharyngeal carcinoma, and non-small-cell lung cancer [47–49].

The tumor microenvironment plays an integral part in various processes of tumorigenesis as well as immune responses [50]. In recent years, the use of ICIs has made irreplaceable achievements in the treatment of BC. Atezolizumab and pembrolizumab targeting PD-1 have been approved as first-line treatment for cisplatin-ineligible metastatic urothelial BC by the Food and Drug Administration [51–54] ‘Cold’ tumor with low T cell infiltration is a critical reason for the resistance to immunotherapy [55,56]. It is essential to determine tumor immune cell infiltration to understand tumor progression and improve the response to immunotherapy. However, no studies have been conducted on the effect of SLC12A8 on the tumor microenvironment. We used CIBERSORT and TIMER 2.0 to analyze the tumor immune cell infiltration of SLC12A8 in bladder cancer. Limitations of various methods to estimate the immune cell composition led to a difference in results. According to CIBERSORT, SLC12A8 may be involved in the complex tumor microenvironment by regulating CD4 + T memory cells, DC cells, macrophages (M0, M1, M2), neutrophils, γ δ T cells, and follicular T-helper cells.

In addition, we found that the expression of SLC12A8 was significantly positively correlated with the expression of common immune checkpoint biomarkers, suggesting that SLC12A8 may inhibit the immune response by inducing the expression of immune checkpoint molecules. Some studies confirmed that high expression of immune checkpoint molecules is associated with a higher response rate. We speculate that the expression of SLC12A8 may h potentially predict the response of immune checkpoint therapy for BC.

In summary, we proposed a prognostic model of SLC12A8 expression level and survival rate in patients with BC. We confirmed overexpression in BC tissues in many databases, clinical samples, and cell lines. More importantly, we first used IHC to analyze SLC12A8 protein expression in normal tissues and bladder tumors, providing evidence for SLC12A8 as a potential diagnostic marker for BC. Furthermore, we found that SLC12A8 may affect the tumor microenvironment and may be a potential marker of immunotherapy response. However, there are some limitations to our study. Our sample size was insufficient and lacked overall survival information. The molecular mechanism of SLC12A8 and research on how it activates the signaling pathway for BC development will be further explored. In addition, we will further investigate the value of SLC12A8 in immunology, such as the effect of SLC12A8 gene expression on immune checkpoint inhibitor response.

### Conclusion

This study confirmed that SLC12A8 was significantly overexpressed in clinical BC tissues using bioinformatics, real-time PCR, and IHC methods. The high expression of SLC12A8 is related to poor outcomes in BC. Subsequently, we found that SLC12A8 was associated with multiple tumor immune cell infiltration and positively correlated with immune checkpoint molecules in BC. SLC12A8 may be a potential biomarker and immunotherapy-related target.

## Data Availability

The original contributions presented in the study are included in the article/Supplementary Material. Further inquiries can be directed to the corresponding authors.

## Highlights

(1) Bioinformatics analysis and clinical tissue validation were combined to analyze the gene expression of SLC12A8 in BC comprehensively.

(2) The correlation between SLC12A8 and tumor immune cell infiltration was explored.

(3) The prognostic value of SLC12A8 was evaluated, and its correlation with immune checkpoints was analyzed.

## Abbreviations

Solute carrier family 12 member 8 (SLC12A8)

Bladder cancer (BC)

Gene Set Enrichment Analysis (GSEA)

Tumor-infiltrating immune cells (TICs)

Multi Experiment Matrix (MEM)

Programmed cell death protein 1 (PD-1)

Programmed death-ligand 1 (PD-L1)

Cytotoxic T-lymphocyte antigen-4 (CTLA-4)

Lymphocyte activation gene 3 protein (LAG3)

T cells immunoglobulin domain mucin domain protein-3 (TIM3)

T cell immunoglobulin and ITIM domain protein (TIGIT)

Molecular Signatures Database (MsigDB)

Hazard ratio (HR)

Gene Ontology (GO)

Kyoto Encyclopedia of Genes and Genomes (KEGG)Area under the curve (AUC)Hazard ratio (HR)

Receiver operating characteristic (ROC)

Extracellular matrix (ECM)

Immune checkpoint inhibitors (ICIs)
